# Integrative Evaluation of Oxidative Changes, Microbial Dynamics, and Quality Deterioration in Fresh Beef During Refrigerated Storage

**DOI:** 10.3390/foods15101788

**Published:** 2026-05-18

**Authors:** Xinyu Zhang, Tehreem Shahwana, Mengran Cai, Sitong Wei, Zihao Zhang, Huale Li, Hui Zhou, Baocai Xu, Zhaoming Wang

**Affiliations:** 1 School of Food and Biological Engineering, Hefei University of Technology, Hefei 230601, China; xinyuzhang_2024@163.com (X.Z.); shahwanatehreem786@gmail.com (T.S.); 15056531371@163.com (M.C.); 15610949185@163.com (S.W.); 15357304112@163.com (Z.Z.); zhouhui@hfut.edu.cn (H.Z.); baocaixu@163.com (B.X.); 2 Hisense Group Co., Ltd., Qingdao 266071, China; lihuale125@163.com; 3 Key Laboratory for Animal Food Green Manufacturing and Resource Mining of Anhui Province, Hefei University of Technology, Hefei 230601, China

**Keywords:** fresh beef, quality deterioration, lipid oxidation, myoglobin autoxidation, protein oxidation, microbial spoilage

## Abstract

The temporal relationship between oxidative processes and microbial succession during aerobic cold storage has not been quantitatively characterized. We hypothesized that oxidation dominates early-stage quality loss, while microbial proliferation drives later-stage deterioration, with the two processes being temporally associated. This study investigated the patterns and mechanisms of quality deterioration in fresh beef stored under aerobic conditions at 4 °C for 10 days. Through comprehensive analysis of microbial communities (via high-throughput sequencing, total viable counts, and TVB-N), oxidation indicators (including TBARS, carbonyl compounds, sulfhydryl content, MetMb content, etc.), and quality characteristics (color, texture, and flavor), strong temporal and quantitative associations were observed. Early-stage quality loss was primarily associated with oxidative processes, as evidenced by a rapid decrease in redness. Meanwhile, later-stage deterioration, particularly flavor and texture decline, was strongly correlated with microbial proliferation, notably the dominance of Pseudomonas spp. Pearson’s correlation analysis revealed that lipid and protein oxidation were significantly associated with microbial total viable counts and changes in key volatile spoilage markers. The results indicate that oxidative and microbial activities are closely associated with beef quality decline, with oxidation mainly associated with color deterioration and microbial activity more strongly associated with flavor changes. These findings demonstrate close associations between co-oxidation processes, microbial succession, and beef quality deterioration, providing a quantitative basis for developing targeted, stage-specific preservation strategies.

## 1. Introduction

China is the world’s third-largest producer of beef [1]. Beef, valued for its distinct nutritional profile (packed with premium protein, essential amino acids, and micronutrients) and palatability, maintains broad consumer appeal. Driven by rising health consciousness, demand for high-quality fresh beef has increased significantly in recent years. As a high-protein commodity, beef freshness critically influences consumer acceptance and commercial value. Nevertheless, post-mortem biochemical processes, such as oxidative degradation and microbial proliferation [2], induce color browning, texture softening, and flavor deterioration, thereby substantially reducing shelf life. Given heightened consumer expectations for meat freshness, elucidating the mechanisms underlying deterioration has emerged as a critical research priority within food science and the meat industry.

Research demonstrates that beef quality deterioration arises from closely associated interactions between biochemical oxidation and microbial metabolism. Kumar et al. [3] observed that during cold storage of beef, light and aerobic conditions exacerbated myoglobin oxidation induced by 4-hydroxy-2-nonenal—a compound derived from lipid oxidation. This exacerbation promoted a significant increase in (metmyoglobin) MetMb formation, which subsequently caused beef discoloration and impaired its sensory quality. Bhandari et al. [4] found that during the repeated freeze–thaw and cold storage of beef, the rapid oxidation of myoglobin and sarcoplasmic proteins resulted in elevated MetMb levels in beef, leading to browning.

Additionally, biochemical changes such as lipid peroxidation and protein carbonylation compromise cellular membrane integrity and generate reactive compounds (e.g., malondialdehyde), which are produced during oxidation and significantly reduce beef quality. These oxidation products have been reported to be closely associated with microbial metabolic activity, suggesting a potential interaction that may influence overall beef quality deterioration. The dominant spoilage bacteria accelerate proteolysis and lipolysis in muscle tissue through extracellular enzyme secretion. Zhang, Zhang, and Li [5] reported that *Carnobacterium*, *Lactobacillus,* and *Leuconostoc* modulate beef quality through metabolic pathways. Hwang et al. [6] reported that psychrophilic bacteria and lactic acid bacteria indirectly affect spoilage-related indices of beef during cold storage, thereby inducing beef spoilage. Nakamura et al. [7] examined major beef cuts stored at 0, 2, and 4 °C, and found that specific microbial communities exerted a significant effect on beef quality under different storage conditions. At lower storage temperatures (0 °C), Lactobacillus spp. became the dominant genus, and the proliferation of this bacterial genus was relatively less likely to trigger rapid beef spoilage. In contrast, at higher refrigeration temperatures (e.g., 4 °C), the abundance of Serratia spp., which possess high spoilage activity, was enhanced. Both the quantitative changes and species variations of these microbial communities are potential regulators of beef quality. Synthesizing the existing research findings on the cold storage process, it is evident that alterations in microbial quantity and community structure constitute one of the key factors contributing to beef quality deterioration.

Previous studies have largely examined oxidative processes and microbial spoilage in isolation. While a framework for their interaction has been proposed in rabbit meat [8], direct, quantitative evidence of their integrated dynamics over refrigerated storage in beef is absent. The novelty of the present study lies in simultaneously establishing the temporal hierarchy of quality deterioration and the quantitative linkage between oxidative and microbial variables through multivariate correlation analysis. This integrated approach is crucial for identifying the stage-specific drivers of spoilage during refrigerated storage.

We hypothesized that the deterioration of fresh beef quality is not a random process but follows a sequential pattern, whereby oxidative reactions initiate the early-stage loss of freshness (primarily discoloration), and the resulting products subsequently facilitate a late-stage dominance of specific spoilage microorganisms, which in turn accelerates sensory deterioration. Previous studies suggest that microorganisms and oxidation may be simultaneously involved in meat quality deterioration [8]. However, their individual and combined contributions to beef spoilage have not been clearly isolated. While the present study focuses on establishing temporal associations and quantitative linkages, further investigation into dose–response relationships between specific oxidation products and microbial growth rates, as well as the variability of microbial community responses across different meat matrices, will be necessary to fully elucidate the mechanistic pathways underlying these associations. Thus, the purpose of this study was to systematically characterize the temporal patterns of quality deterioration and to quantify the associations between oxidative, microbial, and sensory changes in fresh beef.

## 2. Materials and Methods

### 2.1. Sample Preparation

The beef *longissimus thoracis et lumborum* muscles were obtained from three Simmental crossbred cattle (age 24–30 months) from a single slaughter batch, processed under standard commercial conditions. The cattle were slaughtered at night, and the carcasses were transported under refrigerated conditions (2–4 °C) to Hefei Feicui RT-Mart Commercial Co., Ltd. (Hefei, China) in the early morning. Both the *longissimus thoracis* and *lumborum* muscles were collected from each carcass. The muscles were purchased immediately upon arrival, transported to the laboratory within 30 min, and trimmed of connective tissue and visible fat. Subsequently, samples were stored at 4 °C for 10 days, with the aim of analyzing samples for quality parameters, oxidation status, and microbial counts. Beef sampled on the day of purchase was considered the baseline (Day 0). The samples were allocated into six groups (each weighing approximately 500 g). Each storage time point consisted of three independent biological replicates, with each analysis conducted in technical triplicate unless otherwise stated. Each sample was then placed on polypropylene trays and sealed with PVC overwrap (thickness: 0.04 mm; MIAOJIE, Wuxi, China). The PVC overwrap (oxygen transmission rate approximately 10,000–15,000 cm^3^/m^2^/24 h/atm) maintained an aerobic environment critical for both oxidative reactions and the proliferation of aerobic spoilage organisms such as *Pseudomonas* spp. Groups 1 to 6 were analyzed on days 0, 2, 4, 6, 8, and 10, respectively. All reagents were obtained from Sinopharm Chemical Reagent Co., Ltd., Shanghai, China.

### 2.2. Color

Surface color of beef samples was measured according to Chen, Wang, Li, and Xu using a handheld colorimeter (TS7600; 3nh, Shenzhen, China) [9]. To ensure accuracy, the instrument was first standardized with a black-and-white calibration tile before measurement. Then, a smooth and undamaged area was selected for each sample as the testing point, and the three chromaticity parameters of L* (lightness), b* (yellowness), and a* (redness) were recorded. Each sample was measured in triplicate, from which the mean value for each treatment group was derived.

### 2.3. Texture Measurement

A TA-XT plus Texture Analyzer (Stable Micro Systems, Godalming, UK) equipped with a P/36R cylindrical probe was used to perform texture profile analysis (TPA), following a protocol adapted from Wang et al. [10]. Approximately 2 cm^3^ cubes were prepared from the samples and oriented under the probe such that compression was applied perpendicular to the muscle fiber direction. The following settings were applied: a trigger threshold of 5 g, 40% strain, and approach/compression/return speeds of 1, 5, and 5 mm/s, respectively, with a 5 s dwell time between compressions, and measurements were recorded.

### 2.4. Volatile Compound Analysis by GC-IMS

GC-IMS (Gas chromatography-ion mobility spectrometry) was used to profile volatile compounds, following a method adapted from Ma et al. [11]. Briefly, headspace generation was performed by incubating 2.0 g samples in sealed 20 mL vials at 60 °C for 15 min with 500 rpm agitation. Subsequently, an automatic injection of 500 µL headspace vapor was performed (injector: 85 °C) employing a GC-IMS system (FlavorSpec^®^; G.A.S., Dortmund, Germany). A 60 °C isothermal WAX column (30 m × 0.53 mm; Restek, Bellefonte, PA, USA) was employed for segregation. Over a total GC run time of 30 min, high-purity nitrogen (99.99%) was utilized as carrier gas with the following flow rate gradient: initiated at 2 mL/min (12 min), held (8 min), then raised to 10 mL/min (10 min), and finally to 100 mL/min (10 min). Using VOCal software (v1.0.7, G.A.S; G.A.S., Dortmund, Germany) in positive ion mode, data collection was performed with the drift tube maintained at 45 °C and a drift gas (N_2_) flow rate of 150 mL/min. Compound identification was based on spectral matching (NIST/IMS libraries) and validated by retention indices (RI) calibrated with n-ketones C4–C9. The signal intensity for relative quantification was based on the corresponding peak volume.

### 2.5. Peroxide Value (POV)

According to Wang et al. [10], 10 g of minced beef was mixed with 20 mL of a chloroform/methanol mixture (1:1, *v*/*v*) for POV determination. The sample was then homogenized (3000 rpm, 60 s), and 6.16 mL of 0.5% NaCl solution was added. Following vortexing (30 s), the mixture was subsequently centrifuged (1800× *g*, 4 °C, 6 min). After centrifugation, (5 mL) liquid from the bottom chloroform layer was transferred to a glass tube using a glass syringe. Subsequently, 3 mL of a 1:1 chloroform/methanol mixture was added, followed by sequential addition of 100 μL each of ammonium thiocyanate and FeCl2 solutions at concentrations of 3.94 M and 18 mM, respectively. After vortexing (10 s) and incubation (25 °C, 20 min), the absorbance was measured at 500 nm.

### 2.6. Thiobarbituric Acid Reactive Substances (TBARS)

As per the protocol demonstrated by Chen, Wang, Li, and Xu [9], 3 g of minced beef were subjected to homogenization in 30 mL of a trichloroacetic acid-based solution (constituting 7.5% TCA and 0.1% EDTA-2Na) at 10,000 rpm for 60 s to perform extraction. The homogenate was then maintained for 30 min at 50 °C, shaking in a water-bath constant-temperature shaker (Model WE-3, Tianjin Ounuo Instrument Co., Ltd., Tianjin, China). After filtration, (5 mL) filtrate was taken, to which an equal volume of trichloroacetic acid solution was added. It was then heated for 30 min in a water bath at 90 °C. The mixture was cooled to 20 °C upon completion of the reaction, and the absorbance was recorded with a microplate reader (Multiskan FC; Thermo Fisher Scientific, Waltham, MA, USA) at 532 nm.

### 2.7. Metmyoglobin (MetMb) Content

MetMb was determined according to the method described by Wang et al. [8]. Briefly, (5 g) samples were homogenized with 25 mL of 40 mM phosphate buffer (pH 6.8) for 30 s. The homogenate was then centrifuged at 3000 rpm for 30 min at 4 °C. The supernatant was filtered, and absorbance values were measured at 582, 557, 525, and 503 nm using a spectrophotometer (TU-1901; Persee General, Beijing, China).

### 2.8. Soret Band

Soret band analysis was performed according to Chen, Wang, Li, and Xu [9]. The myoglobin solution (prepared per Section 2.7) was subjected to spectral scanning between 380 and 450 nm on a UV-16001 spectrophotometer (TU-1901; Persee General, Beijing, China) at a scanning rate of 800 nm/min.

### 2.9. Heme Iron

By following a slightly modified methodology from Wang et al. [10], heme iron content was quantified. Minced beef (4 g) was placed in a glass tube and homogenized using 20 mL of acidified acetone (45:4:1 acetone/water/HCl, *v*/*v*/*v*). After vortexing for 15 s, the tube was sealed and maintained at 20 °C in the dark for 2 h. The mixture was subjected to filtration through qualitative filter paper, and the filtrate was subsequently analyzed for its absorbance at 640 nm.

### 2.10. Carbonyl Content

As described by Wang et al. [12], under ice-water bath conditions, 1 g minced beef sample was homogenized in 10 mL of ice-cold 0.15 M KCl at 3000 rpm for 60 s. The homogenate was aliquoted into two 100 μL portions. Each aliquot was mixed with 1 mL of 20% trichloroacetic acid (TCA) and centrifuged at 5000× *g* for 5 min. Following supernatant removal, the resulting pellet was resuspended in 400 μL of 5% sodium dodecyl sulfate and sonicated for 60 min at 20 °C. One aliquot was then derivatized by adding 800 μL of 3 M HCl containing 0.3% (*w*/*v*) 2,4-dinitrophenylhydrazine (DNPH), whereas the other (blank) received 800 μL of 3 M HCl only. Following 30 min of incubation at 20 °C, proteins were reprecipitated with 400 μL of 40% TCA and centrifuged again under identical conditions. The pellet underwent three sequential washes with 1 mL of an ethanol/ethyl acetate mixture (1:1, *v*/*v*), each including centrifugation at 10,000× *g* for 5 min. The washed pellet was finally resuspended in 1.5 mL of 20 mM NaH_2_PO_4_ (pH 6.5) with 6 M guanidine hydrochloride. The solution was allowed to stand overnight, after which absorbance values at 280 nm and 370 nm were recorded. The calculation of carbonyl content was performed using a molar extinction coefficient of 22,000 M^−1^ cm^−1^.

### 2.11. Sulfhydryl Content

With minor modifications to the procedure outlined by Wang et al. [13], the sulfhydryl content was determined. Accordingly, 0.5 g of minced beef tissue was homogenized in 10 mL of 50 mM phosphate buffer (pH 7.2) at 3000 rpm for 20 s. In total, 1 mL aliquot of the homogenate was mixed with 9 mL of the same buffer containing 6 mM EDTA, 8 M urea, and 0.6 M NaCl. Following centrifugation (10,000× *g*, 15 min, 4 °C), 3 mL of the supernatant was subjected to a 15-min incubation at 40 °C with 40 μL of 50 mM sodium acetate solution containing 10 mM DTNB. The concentration was then derived from the absorbance at 412 nm, applying a molar extinction coefficient of 13,600 M^−1^ cm^−1^.

### 2.12. Total Viable Count (TVC)

TVC was estimated as per the method illustrated by Chen, Wang, Li, and Xu [9]. Samples (10.0 g) were homogenized aseptically in 90 mL of sterile saline (0.9% NaCl) for 30 s. Based on the estimated microbial load, serial decimal dilutions of the homogenate were prepared. From selected dilutions, 0.1 mL aliquots were spread on plate count agar (PCA) and incubated at 37 °C for 48 h for colony enumeration. Data analysis resulted in bacterial counts expressed on a log CFU/g basis.

### 2.13. Total Volatile Basic Nitrogen (TVB-N)

Approximately 5.0 g of the minced beef sample was homogenized in 50 mL of distilled water and filtered. A 10 mL volume of the resulting supernatant was combined with an equal volume of 10 g/L magnesium oxide suspension for subsequent reaction. Finally, the TVB-N concentration was determined using an automatic Kjeldahl nitrogen analyzer (Hanon K9860; Hanon Instruments, Jinan, China). 

### 2.14. High-Throughput Sequencing and Data Analysis

Microbial community profiling was performed according to an adapted protocol [12]. Following storage, DNA was extracted from the beef samples using the specified kit (Fast Pure Soil DNA Isolation Kit, Magnetic Beads; MJYH, Shanghai, China) in strict accordance with the manufacturer’s instructions. Specifically, a 10 g beef sample was blended with 90 mL of sterile peptone-saline water (0.1% peptone, 0.85% NaCl) in a stomacher for 2 min. An aliquot of 30 mL of the resulting homogenate was then centrifuged at 800× *g* (4 °C, 5 min), and following this, 15 mL of the supernatant was subjected to further centrifugation at 12,000× *g* (4 °C, 5 min). DNA concentration was measured using a NanoDrop 1000 spectrophotometer (Thermo Fisher Scientific, Waltham, MA, USA). Post-storage, DNA from beef samples was extracted using the specified kit (Fast Pure Soil DNA Isolation Kit, Magnetic Beads; MJYH, Shanghai, China), adhering strictly to the provided protocol. PCR amplification was targeted to the V3–V4 regions of the 16S rRNA gene, employing the universal primers 338F and 806R (sequences: 5′-ACTCCTACGGGAGGCAGCAG-3′ and 5′-GGACTACHVGGGTWTCTAAT-3′). After quantification and normalization based on fluorometer readings (Quantus™, Promega, Madison, WI, USA) from 2% agarose gels, the PCR amplicons were submitted for Illumina paired-end sequencing (MiSeq PE300/NovaSeq PE250; Illumina, San Diego, CA, USA) through Majorbio Bio-Pharm Technology Co., Ltd. in Shanghai, China. Microbial community analysis was conducted using three independent biological replicates per storage time point.

Following initial processing (quality filtering with fastp v0.19.6 and read merging with FLASH v1.2.11), operational taxonomic units (OTUs) were delineated using UPARSE (v7.1), which also removed chimeric sequences. Using the SILVA 16S rRNA database (v138) with a 70% confidence coefficient, taxonomic identities were assigned via the RDP classifier (v11.5). To assess alpha diversity, metrics such as Shannon, Simpson, ACE, and Chao indices were computed with Mothur (v20.2). Graphical representation of these metrics was created using QIIME (v1.9.1).

### 2.15. Statistical Analysis

All experiments included three independent replicates, each consisting of triplicate samples. Data were analyzed in SPSS (v. 25.0). Significant main effects were identified through one-way ANOVA with Duncan’s post hoc test, using a significance threshold of *p* < 0.05. Duncan’s multiple range test was selected for mean separation due to its sensitivity in detecting differences among multiple storage time points. However, this approach may increase the probability of Type I error, and results should be interpreted accordingly. Additionally, Pearson’s correlation analysis was performed to evaluate the strength and direction of linear relationships among oxidative, microbial, and quality-related parameters across the six storage time points.

In the ANOVA model applied to volatile flavor compounds, sampling time points (0, 2, 4, 6, 8, and 10 days) were treated as fixed effects, while each batch (with three replicates) was incorporated as a random effect. Visualization of the resulting volatile organic compound profiles was achieved by applying the Gallery Plot plugin in VOCal software (v.1.0.7, G.A.S.).

Sequencing data were analyzed using the QIIME2 platform and R. Alpha diversity within samples was assessed using indices such as Chao1 and Shannon, and between-group comparisons were conducted using the Kruskal–Wallis test. To resolve microbial community composition, the relative abundances of bacterial taxa were calculated at the phylum and genus levels, and community bar plots were generated for visual comparison of compositional differences among groups. For all statistical analyses, a significance level of *p* < 0.05 was applied.

## 3. Results

### 3.1. Color, Texture, and Flavor

The color (L*, a*, and b*) and texture (hardness and springiness) parameters of the beef under refrigerated temperature (4°C) for a storage period of 10 days are presented in Table 1. The initial L* and a* values of the beef samples were 42.79 and 17.64, respectively, while these values decreased significantly to 35.55 and 8.09 (*p* < 0.05), respectively, during the 10-day storage period. Notably, a prominent reduction of approximately 57% in a* values was observed from 0 to 4 days to reach a value of 11.17. The b* values increased approximately 40% from 6.93 to 11.04 (*p* < 0.05) after storage for 10 days at 4 °C. Regarding texture, hardness, and springiness decreased throughout the storage. However, a faster decline was noticed in the later stage of storage as compared with the earlier stage.

**Figure 1:**
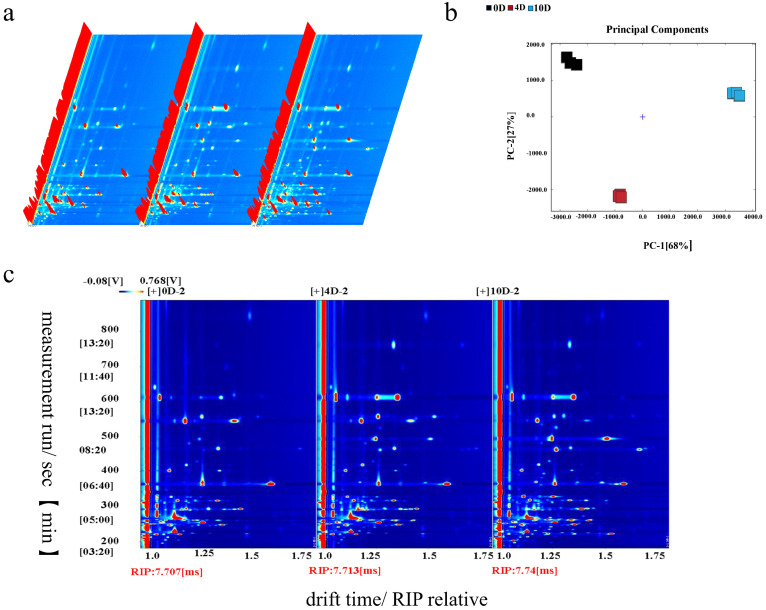
The 3D GC-IMS spectrum of volatile organic compounds in different samples during storage (**a**), the PCA plot of different samples (**b**), and the GC-IMS spectrum for direct comparison of volatile organic compounds in different samples (**c**) were obtained through analysis of variance (ANOVA) and Duncan’s test; each experiment was performed in three independent replicates.

**Figure 2:**
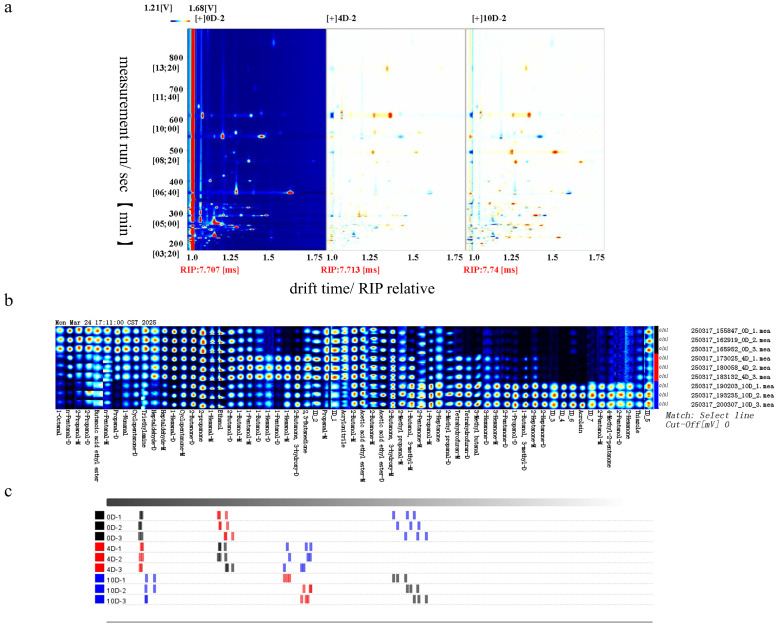
The GC-IMS spectrum for differential comparison of volatile organic compounds in different samples (**a**), the Gallery Plot of volatile organic compounds in different samples (**b**), and the Euclidean distance map between samples (**c**) were obtained through analysis of variance (ANOVA) and Duncan’s test; each experiment was performed in three independent replicates.

Volatile compounds in fresh beef during storage were analyzed using GC-IMS and summarized in Table 2, Figure 1 and Figure 2. Because of the large number of detected compounds, the complete volatile profile is presented in Table 2, with key contributors emphasized in Figure 2. A total of 63 volatile flavor substances were detected across different storage time points, including 14 aldehydes, 17 ketones, 17 alcohols, 3 esters, 3 sulfur- or nitrogen-containing compounds, 3 ethers, and 7 unidentified compounds. During the separation process, some compounds (1-Hexanal, 2-Heptanone, 1-Butanol-3-methyl) were detected in both monomeric and dimeric forms. Aldehydes and ketones are the key flavor compounds in meat. As storage time increased, most aldehydes (heptaldehyde, hexanal, pentanal) exhibited a declining trend, while most ketones (heptanone, hexanone, pentanone) significantly increased. Notably, acrolein, a substance characterized by a markedly pungent odor, accumulated substantially during storage. Among all volatile compounds, thiazole deserves special note. It is a compound with a putrid odor that increased significantly during prolonged storage. In Figure 1b and Figure 2c, the results of PCA and Euclidean distance clearly illustrate the differences in volatile compounds in beef at different storage time points. The results showed a clear separation among the 0-, 4-, and 10-day samples, with flavor profiles diverging most significantly by day 10.

### 3.2. Lipid Oxidation, Myoglobin Autoxidation/Degradation, and Protein Oxidation

**Figure 3:**
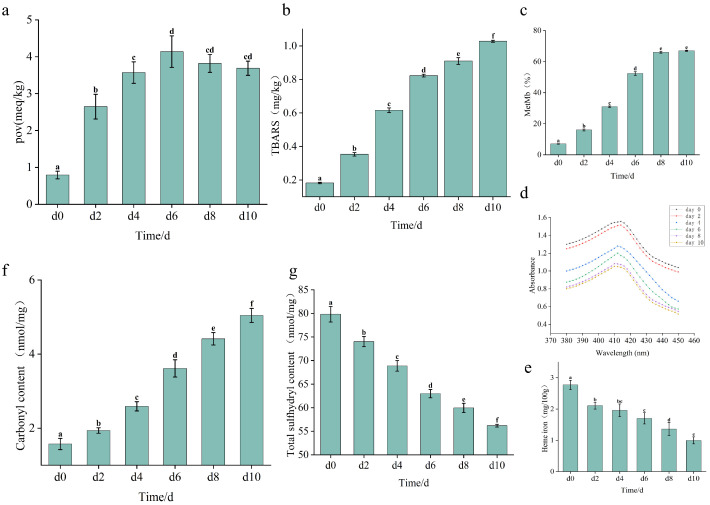
Changes in total sample POV content (**a**), Thiobarbituric Acid Reactive Substances (TBARS) value (**b**), MetMb content (**c**), Soret (**d**), Heme-iron content (**e**), carbonyl content (**f**), and sulfhydryl content (**g**) during storage. The error bars are standard deviations, calculated using ANOVA and Duncan’s test; each experiment was performed in three independent replicates and different lowercase letters (a–f) indicate significant differences (*p* < 0.05).

Figure 3a,b showed the results of lipid oxidation (POV and TBARS), myoglobin autoxidation/degradation (MetMb content), and protein oxidation (carbonyl and Total sulfhydryl content) during beef storage. As shown in Figure 3a, POV increased significantly (*p* < 0.05) during the initial 6 days of refrigerated storage, followed by a non-significant decreasing trend (*p* > 0.05) during prolonged storage. In contrast, TBARS values increased significantly (*p* < 0.05) throughout the entire storage period (Figure 3b). MetMb content exhibited a trend similar to that of POV (Figure 3c). Meanwhile, progressive myoglobin degradation was reflected by changes in the Soret band and heme iron content. The characteristic Soret band absorption peak of myoglobin shifted from 414 nm to 410 nm during prolonged storage of 10 days. Regarding heme iron, its content decreased significantly during refrigerated storage (*p* < 0.05). The data of protein oxidation (Figure 3f,g) displayed the formation of carbonyl compounds and the loss of total sulfhydryl.

### 3.3. TVC, TVB-N, and High-Throughput Sequencing

**Figure 4:**
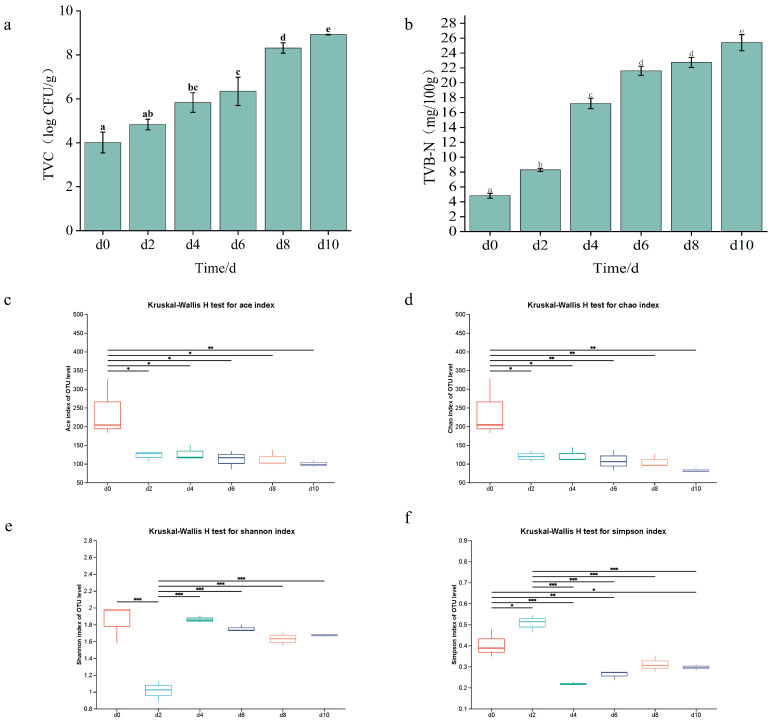
Changes in total sample TVC content (**a**), TVB-N content (**b**), Ace (**c**), Chao (**d**), Shannon (**e**), and Simpson (**f**). The error bars are standard deviations and carried out by ANOVA and Duncan’s test; Each experiment was in three independent replicate experiments. Asterisks indicate significant differences (* *p* < 0.05, ** *p* < 0.01, *** *p* < 0.001). Different lowercase letters (**a**–**f**) indicate significant difference and different colors (**c**–**f**) represent different storage days (Day 0, 2, 4, 6, 8, and 10).

Figure 4a illustrates that the initial TVC value was 3.97 lg CFU/g. However, during refrigeration storage, TVC increased significantly (*p* < 0.05), exceeding the 6 lg CFU/g acceptability threshold by day 6. The result of TVB-N showed a prominent increase (*p* < 0.05) during refrigeration, as shown in Figure 4b. Figure 4c–f depicted the data of ACE, Chao, Shannon, and Simpson index. At day 0, the samples exhibited maximal microbial community richness, while the Simpson index showed an opposite trend. This indicated significantly higher initial diversity in fresh beef. However, at day 10, the elevated Simpson index values indicated microbial diversity, consistent with a reduced microbial community.

**Figure 5:**
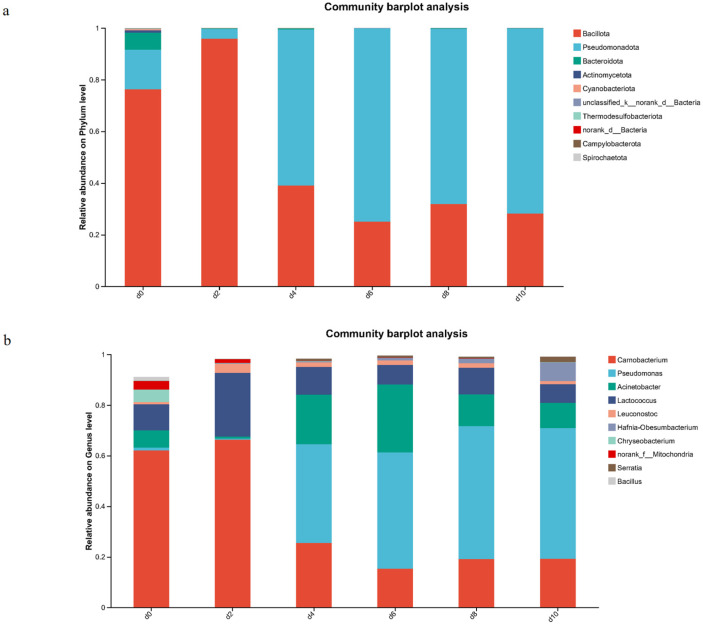
Changes in Bacterial Taxonomic Compositions at Phylum Level (**a**), and Bacterial Taxonomic Compositions at Genus Level (**b**) during Storage. The error bars are standard deviations and carried out by ANOVA and Duncan’s test; Each experiment was in three independent replicate experiments.

This progression highlights temporal divergence in community structure throughout refrigeration. A bar chart visualized the relative abundance of bacterial phyla across samples (Figure 5a). Firmicutes were the dominant phylum, constituted 76% of the total bacterial community during initial refrigeration. With prolonged refrigeration, *Pseudomonadota* (formerly classified under *Proteobacteria*) progressively became predominant, exceeding 70% relative abundance. (Figure 5b) illustrated temporal changes in genus-level bacterial abundance during refrigerated storage. The initial microbial community of beef was dominated by *Carnobacterium* (62.13%), *Lactococcus* (10.30%), *Acinetobacter* (6.76%), and *Chryseobacterium* (4.91%). However, *Pseudomonas* progressively increased, exceeding 50% relative abundance during refrigeration by 10 days.

### 3.4. Correlation Analysis of Oxidative, Microbial, and Quality Indices

To integrate the multi-parametric data and quantify the associations among different deterioration pathways, Pearson’s correlation coefficients were calculated among key physicochemical, oxidative, and microbial indicators across the six storage time points (Days 0, 2, 4, 6, 8, and 10) (Table 3). Lipid oxidation (TBARS) and protein oxidation (carbonyl content) were strongly positively correlated (*p* < 0.01) with microbial growth (TVC) and spoilage markers (TVB-N), with correlation coefficients of r = 0.955 and r = 0.984, respectively. MetMb content showed a strong positive correlation with the relative abundance of Pseudomonas (r = 0.879, *p* < 0.05). Importantly, significant negative correlations were observed between TBARS and desirable quality attributes, including redness (a*; r = −0.967, *p* < 0.01), lightness (L*; r = −0.933, *p* < 0.01), and hardness (r = −0.981, *p* < 0.01). TVC and TVB-N were both strongly negatively correlated with L* (r = −0.988 and r = −0.895, respectively; *p* < 0.01) and hardness (r = −0.991 and r = −0.956, respectively; *p* < 0.01), providing quantitative evidence linking microbial proliferation to the progressive loss of meat quality. Notably, Pseudomonas abundance was significantly correlated with TVB-N (r = 0.942, *p* < 0.01) but not with TVC (r = 0.790, *p* > 0.05), suggesting that the spoilage activity of Pseudomonas may be related more closely to its metabolic output than to total microbial load. These quantitative linkages demonstrate that oxidative processes and microbial communities exhibit highly coordinated behavior during the deterioration of refrigerated beef, with oxidation indices closely tracking microbial proliferation and quality loss across all storage stages.

## 4. Discussion

### 4.1. Integrative Overview of Quality Deterioration Dynamics

Pearson’s correlation analysis (Section 3.4) revealed strong quantitative associations among oxidative reactions, microbial proliferation, and quality deterioration throughout the 10-day storage period. These correlations demonstrate that the three deterioration pathways—lipid and protein oxidation, myoglobin autoxidation, and microbial spoilage—do not progress independently but display highly coordinated behavior. The temporal pattern of these associations indicates that oxidative changes were most closely linked to early-stage discoloration, while microbial indices became increasingly associated with quality decline during later stages. This chronological framework, supported by the correlation matrix, provides the integrative context for the detailed discussion of each deterioration pathway presented below.

### 4.2. Lipid Oxidation, Myoglobin Autoxidation/Degradation, and Protein Oxidation

POV serves as an indicator of hydroperoxide formation during primary lipid oxidation [14]. From the result of POV (Figure 3a), it is concluded that the initial elevation confirms lipid oxidation generating hydroperoxides in refrigerated beef. However, subsequent POV reduction reflects hydroperoxide instability and decomposition into secondary oxidation products, thereby limiting further POV accumulation in later storage phases [15].

MDA, a secondary lipid oxidation product, was quantified through TBARS analysis [16]. Based on established oxidation mechanisms, hydroperoxide decomposition is known to generate secondary oxidation products, including MDA, through pathways such as β-scission of alkoxy radicals. These well-characterized pathways provide a theoretical context for the trends observed, though specific intermediate radicals were not directly measured in this study. Pan et al. [17] described that MDA generation accelerated during later storage stages, coinciding with declining POV, which contrasts with the present study, where MDA exhibited a slower increasing trend during later stages (Figure 3b). This phenomenon potentially results from the concurrent MDA consumption through pathways such as Schiff base formation with proteins during its generation, and both the production and depletion rates of MDA may vary significantly across meat matrices during storage [12].

Myoglobin comprises a globin polypeptide and heme prosthetic group, existing in three redox states: deoxymyoglobin (DeoMb), oxymyoglobin (OxyMb), and metmyoglobin (MetMb), which undergo interconversion [18]. The initial MetMb content accumulation (Figure 3c) confirmed myoglobin autoxidation during refrigeration. The subsequent plateau suggested establishment of redox equilibrium potentially mediated by endogenous oxidants (e.g., H_2_O_2_) that convert MetMb to hyperoxidized species like ferrylmyoglobin [19]. At this stage, MetMb formation and oxidation rates reached dynamic balance, maintaining constant concentrations. The Soret band maximum absorption peak served as a sensitive indicator of heme protein conformational integrity. Progressive reductions in intensity occurred throughout storage, consistent with heme pocket denaturation and porphyrin-globin dissociation as reported by Chen, Wang, Li, and Xu [9]. Overall, these spectral modifications demonstrated concurrent oxidative modification and structural degradation of beef myoglobin during refrigerated storage. Heme iron, a primary form of iron in meat systems, resulted in a progressive intensity reduction throughout storage (Figure 3e). In heme proteins (hemoglobin/myoglobin), iron is normally chelated within the porphyrin macrocycle. Previous studies have shown that Soret band changes during storage are associated with myoglobin degradation followed by the liberation of non-heme iron, thereby reducing measurable heme iron content [20]. Generally, when heme iron converts to non-heme iron, it exhibits potent pro-oxidant activity. It catalyzes lipid and protein oxidation through multiple pathways, including Fenton chemistry and peroxidase-like reactions.

Elevated carbonyl accumulation indicates progressive protein oxidation, as these compounds represent primary autoxidation products formed when ROS modify amino acid side chains [21]. Protein carbonyl formation occurred principally through three mechanisms: metal-catalyzed oxidation of amino acid side chains, advanced glycation end-product formation, and addition with secondary lipid oxidation products. In the present study, lipid-derived carbonyls (particularly aldehydes like MDA and acrolein) promote protein adduct formation during storage, as revealed by the decelerated increasing trend of TBARS in beef during later refrigerated storage stages (Figure 3b). This lipid-protein oxidation interplay has been established across meat systems [22]. Critically, primary lipid oxidation products (hydroperoxides) and secondary derivatives (carbonyls) serve as substrates for protein oxidation, initiating simultaneous degradation upon oxidation [23]. The reduction likely occurs as thiol groups, including surface-exposed free thiols and buried hydrophobic residues, undergo progressive oxidation to form disulfide bonds, thereby decreasing measurable sulfhydryl content [24]. As the most reactive nucleophilic sites in the meat system, thiol groups readily undergo Michael addition reactions with α, β-unsaturated aldehydes derived from lipid oxidation products [25], which also provides an alternative mechanistic perspective for the decelerated accumulation rate of MDA during later storage stages (Figure 3b).

### 4.3. TVC, TVB-N, and High-Throughput Sequencing

TVC serves as a primary microbial acceptability criterion for fresh beef in regulatory standards. The initial TVC value depicted acceptable freshness of raw meat [26]. By day 6, the TVC value exceeded 6 log CFU/g (Figure 4a) and became unacceptable, which was consistent with the findings of Jogdand et al. [27] in emu meat. *Pseudomonas*, *lactic acid bacteria* (*LAB*), and *Enterobacteriaceae* are primary contaminants during slaughtering, processing, and transportation, and they dominate the spoilage microbiota [28]. These organisms promoted proteolytic/lipolytic degradation, resulting in TVB-N accumulation (Figure 4b) and thereby reducing nutritional quality and sensory deterioration. Consequently, TVC quantification provided a critical spoilage indicator, with beef shelf-life directly determined by the composition and load of the spoilage microbiota.

TVB-N quantifies alkaline nitrogenous compounds (ammonia, amines) generated through microbial and enzymatic proteolysis and serves as a major spoilage indicator for meat products [29]. Elevated TVB-N values reflect progressive protein degradation. This accumulation primarily stems from microbial metabolism of spoilage bacteria (*Pseudomonas, Acinetobacter*) and the generation of subsequent volatile amines from endogenous protease activity [30]. Li et al. [31] confirmed specific spoilage organisms drive TVB-N elevation, aligning with our findings (Figure 4b). Concurrently, non-volatile proteolytic products (free amino acids, peptides) contribute indirectly through microbial deamination [32].

Alpha diversity indices characterize bacterial community richness and diversity. ACE and Chao indices (Figure 4c,d) examined OTU richness, while Shannon and Simpson indices quantified species diversity (Figure 4e,f). Alpha diversity analysis revealed high initial microbial diversity in beef, which primarily originated from complex contamination in slaughtering and processing environments [33]. However, microbial diversity significantly decreased during refrigerated storage (Figure 4c–f). This decline was associated with the competitive dominance of specific spoilage microorganisms, leading to a simplification of the community structure. This finding aligns with the pattern of diversity reduction observed in pork by Wang et al. [34], indicating that under the selective pressure of refrigeration, microbial communities tend to be dominated by a few psychrotolerant and highly competitive species, which is a typical characteristic of meat spoilage. The analysis at the phylum and genus levels (Figure 5a,b) clearly demonstrated the succession of dominant bacterial populations. Initially, *Firmicutes* (primarily *Carnobacterium* and *Lactococcus*) were predominant, which are commonly associated with processing environments and have minimal impact on sensory quality at the fresh stage. However, with an extended storage period, the aerobic psychrotolerant *Pseudomonadota* (particularly *Pseudomonas*) proliferated rapidly and became the dominant bacterial group. This succession pattern highly coincides with the results reported by Li et al. [31] in pork, suggesting that this process is commonly observed across different refrigerated red meat systems.

The competitive advantage and spoilage mechanisms of *Pseudomonas* are crucial, as obligate aerobes preferentially utilize glucose in meat products. Upon glucose depletion, they shift to intensive decomposition of proteins and amino acids, producing metabolites such as ammonia, amines, and sulfides. This directly explains the rapid increase in TVB-N values (>15 mg/100 g) exceeding the safety threshold as observed in Figure 4b, resulting in sensory spoilage such as surface mucus, off-odors, and tissue softening [35]. Therefore, the abundance of *Pseudomonas* can be regarded as a key biomarker for predicting the shelf life of beef during refrigerated storage.

While the 16S rRNA amplicon sequencing approach employed in this study effectively resolved the structural succession of the bacterial community during refrigerated storage, it does not directly capture the functional metabolic activities of these microorganisms. The links drawn between Pseudomonas dominance and specific spoilage manifestations—such as TVB-N accumulation and thiazole production—are therefore based on well-established metabolic traits of these taxa reported in the literature, rather than on direct functional measurements. Future studies incorporating metagenomic or metatranscriptomic approaches, together with targeted metabolomics, will be valuable for delineating the specific metabolic pathways through which the spoilage microbiota drive the biochemical and sensory deterioration of refrigerated beef.

### 4.4. Color, Texture, and Flavor

Color serves as a critical indicator of meat freshness, significantly influencing consumers’ initial perceptions. The observed color alterations in beef samples (Table 1) aligned with lipid oxidation (Figure 3a,b), myoglobin autoxidation/degradation (Figure 3c–e), protein oxidation (Figure 3f,g), and microbial spoilage (Figure 4 and Figure 5). Collectively, these findings suggest that beef color alterations are driven by multiple interacting factors. Among these factors, oxidative processes and microbial spoilage appear to positively influence b*, while negatively affecting both L* and a*.

The a* value is a critical color parameter for assessing oxidative effects in meat [36]. OxyMb contributes to the bright red color of fresh beef. During storage, the central heme iron in myoglobin undergoes a transition from ferrous (Fe^2+^) to ferric (Fe^3+^) state (Figure 3c) and the dissociation of the heme group from globin (Figure 3d,e). It resulted in a color shift from bright red to brown. Pearson’s correlation analysis confirmed a strong negative correlation between MetMb content and a* values (r = −0.924, *p* < 0.01), providing quantitative support for this relationship. Along with myoglobin, both lipid and protein oxidation may adversely influence a* values by mediating myoglobin autoxidation. Yang et al. [37] illustrated that lipid oxidation plays a particularly significant role in reducing meat’s redness. Wang et al. [12] also reported that carbonyl compounds derived from protein oxidation promote myoglobin autoxidation. Consistent with these reports, our correlation analysis showed that TBARS and carbonyl content were strongly negatively correlated with a* values (r = −0.967 and r = −0.904, respectively; *p* < 0.01). Lipid oxidation generates several free radicals and α, β-unsaturated aldehydes. The former denatures heme proteins and compromises myoglobin redox stability, while the latter readily binds to myoglobin, facilitating oxidation [8]. The free radical chain reactions of protein oxidation in meat generate numerous alkylperoxyl and thiyl radicals [38], which can modify heme proteins, increasing the instability of myoglobin. Furthermore, existing research indicates that bacterial contamination can promote myoglobin oxidation [39]. Typically, aerobes, specifically *Pseudomonas.* (Figure 5a) decrease the partial pressure of oxygen on the meat surface and facilitate the MetMb formation. In our correlation analysis, Pseudomonas relative abundance was positively correlated with MetMb content (r = 0.879, *p* < 0.05), providing quantitative evidence for this interaction. The aerobic conditions maintained by the PVC overwrap in our storage system are critical for interpreting these results. The low oxygen tension created by Pseudomonas respiration at the meat surface is known to promote MetMb formation [39], directly linking microbial metabolism to myoglobin redox instability. Yang et al. [40] also reported similar results —the growth of *Pseudomonas* fluorescens accelerates the oxidation of DeoMb by drastically reducing the surface oxygen tension through respiratory consumption.

The increase in b* values serves as a direct indicator of meat discoloration [9]. It has been proven that yellowing of beef is closely linked with lipid oxidation [41]. The appearance of yellow pigments in meat products is attributed to non-enzymatic browning reactions between lipid oxidation-derived carbonyls and amino groups present in proteins or phospholipids [42]. In Figure 3b and Table 1, after 6 days of storage, lower MDA contents were associated with lower b* values, further supporting this relationship. Moreover, under oxidative conditions, the accumulation of Schiff base pigments formed from reactions between lipid oxidation products and protein constituents also contributes to yellow discoloration. Therefore, the observed changes in b* values correlate with increases in both TBARS and protein carbonyl contents [8]. Additionally, microbial spoilage during display may influence beef yellowness. Previous studies have reported that various microbial species are capable of producing yellow pigments, such as *xanthomonadins* or those synthesized by Burkholderia cepacia [43]. Certain *Pseudomonas* species have been reported to contribute to green discoloration in meat. However, species-level identification was not confirmed in the present study.

The L* value is used to objectively quantify the lightness of meat surfaces. It is influenced by water migration and alterations in water distribution resulting from muscle microstructure modifications. Generally, the lightness of meat is directly proportional to its free water content. Nawaz et al. [44] reported that high drip loss in meat is closely associated with protein oxidation, indicating that protein oxidation adversely affects the lightness of beef. Furthermore, microbial activity can promote protein degradation, which increases membrane permeability and disrupts meat tissue integrity, ultimately diminishing surface lightness [8]. Moreover, due to the co-oxidation behavior between lipid and protein in muscle food, both myoglobin autoxidation and lipid oxidation can initiate protein oxidation in beef. Studies have demonstrated that myoglobin autoxidation contributes to an undesirable yellow discoloration and a reduction in meat lightness. In summary, lipid oxidation, protein oxidation, myoglobin autoxidation/degradation, and microbial growth directly, indirectly, or jointly influence the color parameters of beef.

Texture is a critical determinant of meat-eating quality for consumers. The result of this study, that both hardness and elasticity decreased throughout storage (Table 1), illustrated trends similar to those reported by Wang et al. [10] in rabbit meat. Hardness is primarily affected by the denaturation and degradation of muscle proteins. On the one hand, Pseudomonas secretes proteases that directly hydrolyze myofibrillar proteins, including collagen and actin, leading to structural loosening. Generally, protein oxidation involves both cross-linkage and degradation. However, the former is responsible for hardness, whereas the latter results in softening. Wang et al. [10] highlighted in their study that any reactive species capable of forming a protein thiyl radical can also induce protein degradation, a phenomenon also termed “polarity reversal catalysis”. Lipid oxidation, myoglobin autoxidation, and protein oxidation can give a wider range of peroxyl, alkoxyl, and hydroxyl radicals that act as electrophiles and abstract hydrogen atoms from C–H bonds, forming carbon-centered radicals. These radicals yield alkyl peroxyl radicals by subsequently reacting with molecular oxygen. These mechanisms, including polarity reversal catalysis and radical-mediated peptide backbone cleavage, have been widely reported in meat systems, although they were not directly quantified in the present study. Through α-amidation pathways, such radicals decompose, leading to peptide backbone cleavage [45].

Flavor in meat products, a critical determinant of consumer preference, is defined as the combined olfactory and oral sensations elicited by lipids and their degradation precursors (e.g., carbohydrates, amino acids, etc.). Flavor development is intrinsically linked to lipid oxidation. During this process, fatty acids degrade by oxidation, sequentially generating primary, secondary, and tertiary oxidation products [46]. The primary products consist predominantly of non-volatile and odorless hydroperoxides [47]. These unstable compounds decompose into a variety of secondary lipid oxidation products, such as aldehydes, alkanes, alcohols, esters, and epoxides, many of which are volatile and significantly influence sensory attributes [48]. Being highly reactive, these secondary products can undergo further degradation to form tertiary oxidation products, either independently or via interactions with other compounds [49]. Beef is particularly prone to generating substantial amounts of secondary oxidation products during storage [50]. In this study, it seems that flavor compounds such as aldehydes, ketones, and alcohols were derived from secondary lipid oxidation products, as revealed by the simultaneous accumulation of MDA (Figure 3b) and acrolein (Table 2). Lee et al. [51] highlighted that aldehydes, ketones, and alcohols are significant contributors to the development of beef aroma. Aldehydes, due to their low odor thresholds (0.07–5.00 μg/kg, except for pentanal at 12–42 μg/kg), play a vital role in beef aroma because of their strong olfactory impact and high volatility [52]. In our work, the relative content of most aldehydes gradually decreased (Table 2), illustrating the loss of the typical flavor of beef. Furthermore, ketones were reported as the second most essential volatile flavor compounds. Interestingly, the relative content of most ketones increased (Table 2). Considering the combined impact of aldehydes and ketones on the flavor of fresh beef, our results suggested that aldehydes appear to be the key contributors to the “freshness” of beef. As storage time increased, aldehydes may transform into ketones, leading to a loss of freshness in the beef. At the late stage of storage, acrolein with a pungent odor accumulates abundantly, indicating that lipid oxidation has exerted an adverse effect on flavor. As previously discussed regarding the co-oxidation of lipid and protein, it was observed that myoglobin autoxidation and protein oxidation may also influence the changes in flavor compounds through their impact on lipid oxidation.

Regarding the relationship between microbial succession and volatile compound changes, volatile data were collected at three time points (Days 0, 4, and 10), which precluded their inclusion in the primary correlation matrix (*n* = 6) used for other variables. Nevertheless, a clear temporal coordination was observed: as *Pseudomonas* relative abundance increased markedly from Day 4 to Day 10 (from below 10% to over 50% of the total community; Figure 5b), the foul-smelling compound thiazole concurrently accumulated (from 90.85 to 184.31; Table 2), while desirable aldehydes such as hexanal progressively declined. This temporal pattern is consistent with the known metabolic characteristics of Pseudomonas spp., which produce sulfur- and nitrogen-containing odor compounds through proteolytic and lipolytic activities [53]. Undoubtedly, microorganisms exert a significant influence on flavor compounds during the storage period of beef. Microorganisms produce alcohols, aldehydes, esters, volatile fatty acids, and sulfur-containing compounds [53]. For instance, *Pseudomonas* is a key producer of alcohols and aldehydes, while *lactic acid bacteria* are associated with butyric acid formation [54]. However, certain microbial metabolic processes (e.g., Pseudomonas) can generate off-odor compounds. Notably, our research found that thiazole compounds with foul odors accumulated significantly during the late storage period (Table 2), and this accumulation coincided temporally with the dominance of Pseudomonas in the microbial community, indicating that microorganisms, specifically *Pseudomonas,* have a substantial impact on the flavor of beef in the later stages of storage.

**Figure 6:**
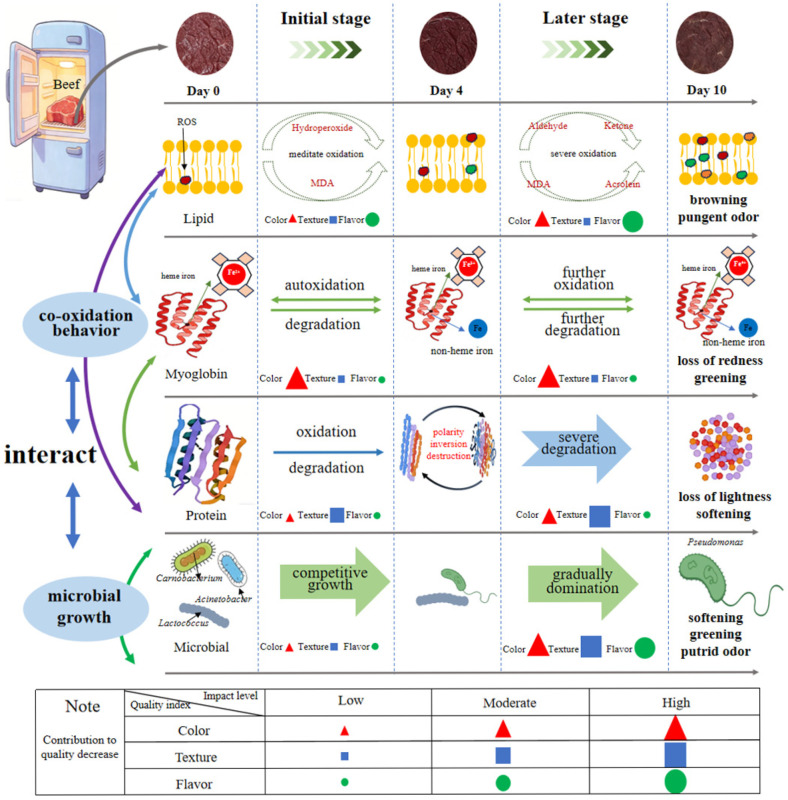
The quality deterioration mechanism of fresh beef under refrigeration temperature.

Figure 6 presents a proposed temporal model of beef quality deterioration under aerobic refrigeration at 4 °C, constructed from the quantitative associations and temporal sequences identified in this study. During storage, the sensory quality characteristics of fresh beef, including color, texture, and flavor, gradually degrade, leading to browning, softening, and off-flavor, among others. Rather than simply listing contributing factors, this model follows a unidirectional cause-and-effect flow: oxidative reactions initiate early-stage quality loss, and the resulting products subsequently facilitate the late-stage dominance of spoilage microorganisms, which, in turn, accelerate sensory deterioration. Specifically, this study identified strong temporal and quantitative associations between oxidation and microbial spoilage, as evidenced by significant Pearson’s correlations between lipid oxidation markers (TBARS) and microbial total viable counts (r = 0.955, *p* < 0.01), and between MetMb content and a* values (r = −0.924, *p* < 0.01). While these associations suggest that oxidative products may be closely linked to microbial metabolism and that aerobic microbial activities (e.g., Pseudomonas) were closely associated with oxidative changes, the present observational design does not establish causality.

Among the quality indicators, browning occurred quickly at the early stage, while texture softening and off-flavor developed at the later stage. This phenomenon is consistent with the oxidation reaction, specifically myoglobin autoxidation, whereas the dominant role of microorganisms is observed at a later stage. Co-oxidation between lipid and protein occurred, while the growth of dominant microorganisms gradually increased throughout the storage period. At the initial stage, the co-oxidation behavior affected redness by influencing myoglobin autoxidation, whereas microorganisms contributed little to beef browning. However, at a later stage, lipid and protein oxidation and microbial spoilage negatively influenced color by certain oxidation products or microbial metabolites, such as the yellow color of MDA affecting b* value and protein degradation affecting L* value. Certain Pseudomonas species have been reported to contribute to green discoloration in meat. However, species-level identification was not confirmed in the present study. Lipid and protein oxidation were correlated with changes in texture and flavor throughout storage. Meanwhile, the impact of microbial spoilage on protein degradation and the generation of pungent off-odors significantly surged at later storage. Compounds such as acrolein (from lipid oxidation) and thiazole (associated with microbial metabolism) were both detected. They likely contributed to the overall off-flavor and pungent smell, although their individual contributions could not be isolated. Similarly, both protein oxidation and microbial growth were associated with protein degradation through distinct mechanisms: oxidative modification and bacterial enzymes, respectively. Given the observational and correlational nature of this study, it remains difficult to distinguish the foremost cause of both flavor and texture degradation; thus, the model identifies a sequence of associations rather than a confirmed causal pathway.

Several limitations of the present study should be acknowledged. The use of a single slaughter batch and a single breed (Simmental crossbred) minimized confounding factors and strengthened the internal consistency of the temporal observations. However, this may limit the generalizability of the findings to other cattle populations, breeds, or production systems. The inherent biological variability among individual animals—in terms of initial microbial load, fat content, enzymatic activity, and antioxidant capacity—could influence the rate and pattern of spoilage. Future studies incorporating a larger number of animals from diverse sources and multiple batches would be valuable to assess the contribution of biological variability to the observed oxidative and microbial dynamics and to confirm the robustness of the identified temporal hierarchy across broader populations. Additionally, the volatile compound data were collected at only three time points (Days 0, 4, and 10), which precluded their inclusion in the primary Pearson’s correlation matrix. Future studies with more frequent volatile sampling would enable a more robust statistical integration of flavor changes with microbial and oxidative variables. Furthermore, the single-temperature (4 °C) observational design cannot establish causal relationships between oxidative and microbial processes; targeted interventional studies using antioxidant or antimicrobial treatments, as well as multi-temperature comparisons, are required to isolate their independent and causal roles in beef quality deterioration.

## 5. Conclusions

This study reveals strong temporal and quantitative associations between oxidative and microbial pathways in beef spoilage. Our findings demonstrate that quality deterioration appeared to follow a sequential pattern, with discoloration occurring prior to significant flavor and texture deterioration. Oxidative changes were strongly correlated with microbial proliferation across all storage stages, as evidenced by significant Pearson’s correlations between TBARS, TVC, and key quality indices. Although oxidative and microbial changes occurred concurrently and showed strong associations with quality deterioration, this study does not isolate their causal direction. Further studies using targeted antioxidant or antimicrobial interventions are required to clarify their independent and causal roles. The insights gained suggest the potential value of integrated preservation strategies that simultaneously target oxidative reactions and microbial growth to effectively extend the shelf-life of fresh beef during refrigerated storage.

## Figures and Tables

**Table 1:** Changes in color and texture during storage.

Index	Time/d	Treatment
L*	0	42.74 ± 0.07 ^a^
	2	40.47 ± 0.16 ^b^
	4	40.17 ± 0.01 ^b^
	6	39.32 ± 0.75 ^c^
	8	36.53 ± 0.28 ^d^
	10	35.56 ± 0.5 ^e^
a*	0	17.64 ± 0.06 ^a^
	2	13.72 ± 0.12 ^b^
	4	11.17 ± 0.03 ^c^
	6	10.45 ± 0.25 ^d^
	8	9.63 ± 0.40 ^e^
	10	8.26 ± 0.46 ^f^
b*	0	6.93 ± 0.02 ^a^
	2	7.37 ± 0.04 ^b^
	4	8.70 ± 0.34 ^c^
	6	9.27 ± 0.18 ^d^
	8	9.75 ± 0.25 ^e^
	10	11.04 ± 0.11 ^f^
Hardness(N)	0	1290.44 ± 15.78 ^a^
	2	1181.73 ± 15.69 ^b^
	4	1075.97 ± 16.52 ^c^
	6	946.83 ± 17.95 ^d^
	8	804.81 ± 14.10 ^e^
	10	705.54 ± 4.44 ^f^
Springiness	0	0.97 ± 0.002 ^a^
	2	0.96 ± 0.003 ^b^
	4	0.96 ± 0.004 ^b^
	6	0.94 ± 0.004 ^c^
	8	0.93 ± 0.005 ^d^
	10	0.89 ± 0.009 ^e^

Significant intra-group and intergroup differences are denoted by different letters (a–f), respectively (*p* < 0.05), analyzed by ANOVA and Duncan’s test; each experiment was replicated three times independently.

**Table 2:** Changes in the content of volatile flavor compounds in beef during storage.

Intensity (V)					
No.	Compound Name	Fomula	D1	D4	D10
Aldehydes (14)					
V1	1-Nonanal	C_9_H_18_O	486.35 ± 39.74 ^a^	360.39 ± 16.90 ^b^	215.68 ± 12.04 ^c^
V2	1-Octanal	C_8_H_16_O	226.46 ± 12.20 ^a^	109.98 ± 7.39 ^b^	91.38 ± 3.14 ^c^
V3	Heptaldehyde-D	C_7_H_14_O	78.43 ± 3.43 ^a^	76.96 ± 8.38 ^b^	46.51 ± 8.92 ^c^
V4	Heptaldehyde-M	C_7_H_14_O	559.45 ± 7.26 ^a^	542.91 ± 26.69 ^b^	346.71 ± 71.32 ^c^
V5	1-Hexanal-D	C_6_H_12_O	3530.12 ± 73.30 ^a^	2884.70 ± 154.40 ^b^	1741.60 ± 342.06 ^c^
V6	1-Hexanal-M	C_6_H_12_O	2194.79 ± 32.77 ^a^	1988.80 ± 43.84 ^b^	1578.22 ± 111.53 ^c^
V7	n-Pentanal-D	C_5_H_10_O	761.90 ± 35.04 ^a^	490.43 ± 39.11 ^b^	119.60 ± 18.16 ^c^
V8	n-Pentanal-M	C_5_H_10_O	456.05 ± 10.23 ^a^	377.51 ± 7.01 ^b^	234.35 ± 16.16 ^c^
V9	Propanal-D	C_3_H_6_O	596.76 ± 47.15 ^a^	407.38 ± 29.16 ^b^	85.00 ± 10.37 ^c^
V10	Propanal-M	C_3_H_6_O	541.82 ± 17.18 ^a^	555.61 ± 8.43 ^b^	242.75 ± 10.66 ^c^
V11	2-Methyl propanal-D	C_4_H_8_O	29.34 ± 4.73 ^a^	37.08 ± 1.22 ^a^	63.54 ± 13.89 ^b^
V12	2-Methyl propanal-M	C_4_H_8_O	115.29 ± 7.14 ^a^	128.33 ± 3.64 ^b^	121.96 ± 7.01 ^ab^
V13	3-Methyl butanal	C_5_H_10_O	62.25 ± 8.73 ^a^	373.24 ± 75.39 ^b^	710.42 ± 174.36^c^
V14	Acrolein	C_3_H_4_O	30.39 ± 0.87 ^a^	25.46 ± 1.73 ^a^	366.53 ± 96.79 ^b^
Ketnoes (17)					
V15	2-Butanone, 3-hydroxy-D	C_4_H_8_O2	517.83 ± 107.74 ^a^	3616.70 ± 184.44 ^b^	2955.46 ± 316.10 ^c^
V16	2-Butanone, 3-hydroxy-M	C_4_H_8_O2	1981.43 ± 146.18 ^a^	2964.94 ± 18.90 ^b^	2940.52 ± 30.17 ^bc^
V17	3-Heptanone	C_7_H_14_O	62.17 ± 2.16 ^a^	73.62 ± 10.45 ^a^	98.67 ± 4.97 ^b^
V18	2-Heptanone-D	C_7_H_14_O	42.47 ± 1.39 ^a^	71.02 ± 0.41 ^b^	594.95 ± 68.66 ^c^
V19	2-Heptanone-M	C_7_H_14_O	144.40 ± 5.86 ^a^	337.07 ± 14.40 ^b^	1078.85 ± 29.90 ^c^
V20	2-Hexanone	C_6_H_12_O	38.95 ± 5.32 ^a^	45.08 ± 7.06 ^a^	86.95 ± 4.20 ^a^
V21	3-Hexanone-D	C_6_H_12_O	89.62 ± 1.01 ^a^	170.10 ± 6.31 ^b^	257.88 ± 25.87 ^c^
V22	3-Hexanone-M	C_6_H_12_O	45.66 ± 1.44 ^a^	106.77 ± 1.56 ^b^	289.05 ± 15.50 ^c^
V23	2-Pentanone-D	C_5_H_10_O	211.38 ± 13.82 ^a^	426.15 ± 17.10 ^b^	1485.98 ± 63.55 ^c^
V24	2-Pentanone-M	C_5_H_10_O	137.21 ± 10.53 ^a^	126.05 ± 11.66 ^a^	298.69 ± 8.25 ^b^
V25	2-Butanone-D	C_4_H_8_O	2608.31 ± 63.07 ^a^	1990.11 ± 44.00 ^b^	2209.70 ± 28.37 ^c^
V26	2-Butanone-M	C_4_H_8_O	479.30 ± 9.39 ^a^	481.21 ± 6.45 ^a^	421.63 ± 7.05 ^b^
V27	Cyclopentanone-D	C_5_H_8_O	196.94 ± 29.11 ^a^	152.75 ± 29.82 ^b^	125.49 ± 18.30 ^b^
V28	Cyclopentanone-M	C_5_H_8_O	629.23 ± 41.09 ^a^	553.45 ± 34.53 ^a^	512.86 ± 31.94 ^b^
V29	2-propanone	C_3_H_6_O	3861.00 ± 80.60 ^a^	3031.89 ± 35.47 ^b^	2876.12 ± 61.27 ^c^
V30	2,3-Butanedione	C_4_H_6_O_2_	350.63 ± 43.92 ^a^	768.24 ± 22.71 ^b^	481.56 ± 30.52 ^c^
V31	4-Methyl-2-pentanone	C_6_H_12_O	21.61 ± 1.07 ^a^	23.04 ± 1.72 ^a^	81.40 ± 5.20 ^b^
V32	Thiazole	C_3_H_3_NS	90.85 ± 13.98 ^a^	126.18 ± 10.03 ^b^	184.31 ± 56.40 ^c^
Alcohols (17)					
V33	1-Hexanol-D	C_6_H_14_O	46.40 ± 7.12 ^a^	129.36 ± 21.46 ^b^	85.63 ± 10.93 ^c^
V34	1-Hexanol-M	C_6_H_14_O	108.45 ± 6.08 ^a^	897.88 ± 95.96 ^b^	687.57 ± 56.18 ^c^
V35	1-Pentanol-M	C_5_H_12_O	615.46 ± 72.90 ^a^	1102.67 ± 26.14 ^b^	476.32 ± 22.84 ^c^
V36	1-Propanol-D	C_3_H_8_O	76.48 ± 3.26 ^a^	123.32 ± 3.03 ^b^	787.69 ± 23.22 ^c^
V37	1-Butanol, 3-methyl-D	C_5_H_12_O	105.01 ± 1.17 ^a^	562.31 ± 11.42 ^b^	2064.01 ± 93.82 ^c^
V38	1-Butanol, 3-methyl-M	C_5_H_12_O	255.81 ± 18.58 ^a^	1040.55 ± 10.18 ^b^	1551.18 ± 18.73 ^c^
V39	1-Butanol-D	C_4_H_10_O	33.06 ± 4.39 ^a^	61.36 ± 3.88 ^b^	27.12 ± 2.40 ^a^
V40	1-Butanol-M	C_4_H_10_O	272.93 ± 9.03 ^a^	414.03 ± 2.89 ^b^	284.53 ± 11.57 ^c^
V41	2-Butanol-D	C_4_H_10_O	440.95 ± 19.90 ^a^	499.33 ± 13.91 ^b^	368.14 ± 49.17 ^c^
V42	2-Butanol-M	C_4_H_10_O	508.54 ± 10.74 ^a^	480.84 ± 8.79 ^b^	462.43 ± 18.47 ^c^
V43	2-Pentanol-D	C_5_H_12_O	10.43 ± 2.85 ^a^	9.95 ± 1.33 ^a^	26.18 ± 3.29 ^b^
V44	2-Pentanol-M	C_5_H_12_O	30.87 ± 0.21 ^a^	30.30 ± 5.00 ^a^	122.63 ± 9.19 ^b^
V45	1-Propanol-D	C_3_H_8_O	76.48 ± 3.26 ^a^	123.32 ± 3.03 ^b^	787.69 ± 23.22 ^c^
V46	1-Propanol-M	C_3_H_8_O	333.20 ± 8.57 ^a^	426.64 ± 9.37 ^b^	852.04 ± 9.32 ^c^
V47	2-Propanol-D	C_3_H_8_O	280.06 ± 8.15 ^a^	167.90 ± 19.01 ^b^	184.96 ± 62.65 ^c^
V48	2-Propanol-M	C_3_H_8_O	228.21 ± 1.35 ^a^	119.08 ± 14.34 ^b^	66.37 ± 13.32 ^c^
V49	Ethanol	C_2_H_6_O	5989.67 ± 82.04 ^a^	5810.06 ± 64.42 ^b^	4213.47 ± 65.58 ^c^
Esters (3)					
V50	Acetic acid ethyl ester-D	C_4_H_8_O_2_	624.94 ± 17.40 ^a^	718.10 ± 28.46 ^b^	677.57 ± 106.53 ^c^
V51	Acetic acid ethyl ester-M	C_4_H_8_O_2_	554.21 ± 7.29 ^a^	597.01 ± 9.26 ^b^	596.74 ± 30.86 ^b^
V52	Butanoic acid ethyl ester	C_6_H_12_O_2_	77.13 ± 1.79 ^a^	60.19 ± 1.88 ^b^	78.13 ± 1.98 ^a^
Others (11)					
V53	Acrylonitrile	C_3_H_3_N	136.84 ± 6.53 ^a^	145.80 ± 7.49 ^ab^	103.20 ± 2.83 ^c^
V54	Triethylamine	C_6_H_15_N	121.20 ± 3.48 ^a^	81.40 ± 1.59 ^b^	82.77 ± 3.81 ^b^
V55	Tetrahydrofuran-D	C_4_H_8_O	13.49 ± 1.35 ^a^	72.27 ± 8.93 ^b^	139.44 ± 4.10 ^c^
V56	Tetrahydrofuran-M	C_4_H_8_O	55.50 ± 4.74 ^a^	208.84 ± 9.75 ^b^	281.29 ± 4.93 ^c^
V57	ID_1	*	55.88 ± 3.96 ^a^	58.93 ± 5.71 ^a^	40.69 ± 2.77 ^b^
V58	ID_2	*	229.04 ± 5.42 ^a^	305.16 ± 14.53 ^b^	262.83 ± 40.62 ^c^
V59	ID_3	*	6.58 ± 0.70 ^a^	6.49 ± 0.61 ^a^	69.11 ± 10.40 ^b^
V60	ID_4	*	37.86 ± 1.52 ^a^	28.85 ± 5.21 ^b^	750.71 ± 76.61 ^c^
V61	ID_5	*	196.21 ± 1.90 ^a^	145.98 ± 4.73 ^b^	210.67 ± 4.45 ^c^
V62	ID_6	*	28.47 ± 3.20 ^a^	10.02 ± 1.18 ^b^	177.38 ± 16.29 ^c^
V63	ID_7	*	12.04 ± 2.16 ^a^	10.65 ± 0.70 ^a^	74.63 ± 10.93 ^b^

Significant intra-group and intergroup differences are denoted by different letters (a–c), respectively (*p* < 0.05), analyzed by ANOVA and Duncan’s test; each experiment was replicated three times independently. * indicates unidentified substances for which no chemical formula is available.

**Table 3:** Pearson’s correlation coefficients among oxidative, microbial, and quality indices of fresh beef during refrigerated storage at 4 °C for 10 days.

	TBARS	Carbonyl	Sulfhydryl	MetMb	TVC	TVB-N	Pseudomonas	L*	a*	b*	Hardness	Springiness
TBARS	1											
Carbonyl	0.974 **	1										
Sulfhydryl	−0.997 **	−0.982 **	1									
MetMb	0.986 **	0.985 **	−0.986 **	1								
TVC	0.955 **	0.984 **	−0.967 **	0.965 **	1							
TVB-N	0.994 **	0.949 **	−0.983 **	0.971 **	0.927 **	1						
Pseudomonas	0.906 *	0.833 *	−0.869 *	0.879 *	0.79	0.942 **	1					
L*	−0.933 **	−0.965 **	0.956 **	−0.942 **	−0.988 **	−0.895 *	−0.712	1				
a*	−0.967 **	−0.904 *	0.966 **	−0.924 **	−0.909 *	−0.966 **	−0.848 *	0.911 *	1			
b*	0.979 **	0.978 **	−0.980 **	0.957 **	0.967 **	0.967 **	0.871 *	−0.941 **	−0.937 **	1		
Hardness	−0.981 **	−0.996 **	0.990 **	−0.984 **	−0.991 **	−0.956 **	−0.828 *	0.979 **	0.933 **	−0.982 **	1	
Springiness	−0.870 *	−0.937 **	0.897 *	−0.866 *	−0.921 **	−0.826 *	−0.66	0.925 **	0.807	−0.933 **	0.932 **	1

Significant correlations are indicated by asterisks: * *p* < 0.05; ** *p* < 0.01.

## Data Availability

The original contributions presented in this study are included in the article. Further inquiries can be directed to the corresponding author.
